# Use of 16S ribosomal RNA gene analyses to characterize the bacterial signature associated with poor oral health in West Virginia

**DOI:** 10.1186/1472-6831-11-7

**Published:** 2011-03-01

**Authors:** Joan C Olson, Christopher F Cuff, Slawomir Lukomski, Ewa Lukomska, Yeremi Canizales, Bei Wu, Richard J Crout, John G Thomas, Daniel W McNeil, Robert J Weyant, Mary L Marazita, Bruce J Paster, Thomas Elliott

**Affiliations:** 1 Department of Microbiology, Immunology and Cell Biology, West Virginia University, Morgantown, WV, USA; 2 Health Effects Laboratory Division, CDC, NIOSH, Morgantown, WV, USA; 3 School of Dentistry, West Virginia University, Morgantown, WV, USA; 4 School of Nursing, Duke University Medical Center, Durham, NC, USA; 5 Department of Periodontology, School of Dentistry, West Virginia University, Morgantown, WV, USA; 6 Department of Pathology, West Virginia University, Morgantown, WV, USA; 7 Department of Clinical Psychology and Clinical Professor of Dental Practice and Rural Health, West Virginia University, Morgantown, WV, USA; 8 Department of Dental Public Health and Information Management, University of Pittsburgh, School of Dental Medicine, Pittsburgh, PA, USA; 9 Center for Craniofacial and Dental Genetics, Department of Oral Biology, School of Dental Medicine, University of Pittsburgh, Pittsburgh, PA; 10 Department of Human Genetics, Graduate School of Public Health, University of Pittsburgh, Pittsburgh, PA, USA; 11 Department of Molecular Genetics, The Forsyth Institute, Cambridge, MA; 12 Department of Oral Medicine, Infection and Immunity, Harvard School of Dental Medicine, Boston, MA, USA

## Abstract

**Background:**

West Virginia has the worst oral health in the United States, but the reasons for this are unclear. This pilot study explored the etiology of this disparity using culture-independent analyses to identify bacterial species associated with oral disease.

**Methods:**

Bacteria in subgingival plaque samples from twelve participants in two independent West Virginia dental-related studies were characterized using 16S rRNA gene sequencing and Human Oral Microbe Identification Microarray (HOMIM) analysis. Unifrac analysis was used to characterize phylogenetic differences between bacterial communities obtained from plaque of participants with low or high oral disease, which was further evaluated using clustering and Principal Coordinate Analysis.

**Results:**

Statistically different bacterial signatures (*P *< 0.001) were identified in subgingival plaque of individuals with low or high oral disease in West Virginia based on 16S rRNA gene sequencing. Low disease contained a high frequency of *Veillonella *and *Streptococcus*, with a moderate number of *Capnocytophaga*. High disease exhibited substantially increased bacterial diversity and included a large proportion of Clostridiales cluster bacteria (*Selenomonas*, *Eubacterium, Dialister*). Phylogenetic trees constructed using 16S rRNA gene sequencing revealed that Clostridiales were repeated colonizers in plaque associated with high oral disease, providing evidence that the oral environment is somehow influencing the bacterial signature linked to disease.

**Conclusions:**

Culture-independent analyses identified an atypical bacterial signature associated with high oral disease in West Virginians and provided evidence that the oral environment influenced this signature. Both findings provide insight into the etiology of the oral disparity in West Virginia.

## Background

West Virginians have the worst oral health in the nation, with almost twice the national average (48.2%) of adults aged 65 or more having all their natural teeth extracted [1]. These statistics become more alarming knowing that infections of the oral cavity have been associated with chronic diseases, such as diabetes, cardiovascular disease and atherosclerosis [2-4]. Neither the origin of poor oral health in West Virginia nor its relationship with systemic disease is understood. Central to this problem is a determination of the microbial populations responsible for oral infections. Historically this has been difficult because of the complexity of the microbiome within oral biofilms, and difficulties in cultivating bacteria obtained from the oral environment.

Biofilms can play either a protective (probiotic) or pathogenic role in oral health depending upon their microbial composition. Oral biofilms are initiated by colonization of probiotic Gram-positive cocci, primarily streptococci, adhering to the tooth surface, along with coaggregating *Actinomyces *and *Veillonella *[5]. Coaggregation is a common property in plaque development, and early colonizing bacteria are bridged through bacteria, such as fusobacteria, to late colonizers. The ecological succession of microbial populations from early colonizing Gram-positive cocci to late colonizing Gram-negative anaerobes of diverse morphotypes leads to a shift in biofilm composition that correlates with the appearance of gingivitis and periodontitis [6]. Specific organisms have been linked with oral diseases. Dental caries occurs as a result of a shift in the biofilm community towards acidogenic and acid-tolerant bacteria, specifically *Streptococcus mutans *and lactobacilli [7]. In subgingival plaque, *Porphyromonas gingivalis, Tannerella forsythia *and *Aggregatibacter actinomycetemcomitans *have been strongly associated with periodontal disease [8,9]. Until recently, associations of microbes with oral disease have been based on in vitro cultivation. As it is now recognized that only about 60% of the species in oral biofilms are cultivable [10], the use of culture-independent analyses has led to a new level of understanding of oral associated microbes [11].

Molecular analyses of periodontal microflora had not previously been used to examine the bacterial profile of subgingival plaque of West Virginians. The goal of this study was to use 16S rRNA gene analyses to gain insight into the etiology of the oral health disparity observed in this population. In this preliminary study we were able to identify significantly different 16S rRNA bacterial phylogenetic signatures in plaque from individuals having high or low oral disease, and the high disease signature was evident in two independent populations that span a wide range of age groups. Overall we found that communities rich in *Veillonella *and streptococci shifted to communities rich in *Selenomonas *and other Clostridiales in association with a decline in oral health, potentially linking an atypical bacterial signature with oral disease in West Virginians. The finding that an atypical bacterial signature may be linked to oral health disparities observed in West Virginia highlights the need for further analyses of bacterial species associated with high and low oral disease in this population in order to understand the origin of this disparity.

## Methods

### Subject populations

Subgingival plaque samples used in this study were obtained in conjunction with two research projects conducted in West Virginia. Plaque from an age 23 to 48 population was obtained through the Center for Oral Health Research in Appalachia (COHRA study), a partnership between West Virginia University and University of Pittsburgh examining genetic, epidemiologic, microbiological, and behavioral factors contributing to oral health. Plaque from an older population was obtained through the Oral Health Disparities Among Elders With and Without Cognitive Impairment (Cognitive study), performed in collaboration with the West Virginia University School of Dentistry and the Center on Aging. We chose to include subjects from two independent West Virginia population pools (median age 23-48 or age >70) in our study to help extend an understanding of the relevance of our findings to a broad sample of the West Virginian population. Being funded as a pilot project, relatively few subjects from each group were able to be analyzed. All procedures were performed using Institutional Review Board approved protocols.

### Subject evaluations

Criteria for oral health evaluation, methods of plaque collection from participants, and calibration of researchers in the COHRA project have been previously described [12]. Our study examined subgingival plaque from seven COHRA participants, which was obtained from four first molars (#3, 14, 19 and 30). These same sites were assessed for probing depth (PD), recession and bleeding on probing (BOP), as summarized in Table 1. Caries assessment was based on the coronal tooth surfaces, and teeth were classified as sound, decayed, filled or missing. Percent sound teeth for each participant is reported in Table 1. The oral status of COHRA study subjects was determined based on periodontal examinations (bordered by dashed lines in Table 1): low disease defined as <3.5 mm PD, 0-25% BOP sites; high disease defined as >4.5 mm PD, 100% BOP sites. Subgingival plaque was sampled using a curette, suspended in 100-500 μl TE (10 mM Tris, pH 7.5, 1 mM EDTA) containing 20% glycerol and stored at -70°C until processing. Multiple specimens from individual participants were pooled for analyses.

**Table 1:** COHRA study clinical evaluations

Patient code	DB	DC	DF	DL	DG	DI	DA
Age	30 y	23 y	32 y	32 y	48 y	40 y	35 y
Sex	F	F	M	F	M	M	F
Race	White	Afr/Am	White	White	Mixed	White	White
Smoker	No	No	Sometimes	No	Yes	Yes	Sometimes
							
**Periodontal ^1^**							
(Measured at 4 sites: 3,14,19,30)							
Probing depth (mean mm/site)	< 3.5	< 3.5	4.5	4.5	5.2	5.2	5.2
Recession (% positive/site)	0	0	0	25	0	75	100
Bleeding on probing (% of sites)	25	0	100	100	100	100	100
Gingivitis - localized	0	0	0	0	0	0	0
generalized	0	Yes	Yes	0	0	0	0
hyperplasia	0	0	0	Yes	Yes	Yes	Yes
							
**Caries**							
Dentures (lower/upper)	0	0	0	0	0	0	0
Sound teeth (% of 28 teeth)	64.3	78.6	92.9	53.6	78.6	67.9	50

^1 ^Dashed lines border clinical parameters used to determine the oral status of COHRA study participants.

Cognitive study participants were selected based on: 1) age 70 years and older, 2) resident of West Virginia, 3) community-living, and 4) dentate (having at least four natural teeth). Oral evaluations were performed by calibrated researchers using guidelines and protocols from the National Health and Nutrition Examination Survey (NHANES IV) [13]. Subgingival plaque samples were collected using sterile periodontal curettes from six sites as follows: the buccal surface of the most anterior molar in each quadrant, and the buccal surface of #11 and #31. Plaque samples were stored and pooled for analyses as above. Criteria for periodontal evaluation in the Cognitive study included PD, gingivitis and calculus. Caries evaluation included type of dentures and percent sound, missing, filled, decayed or crowned teeth. Both evaluations are reported in Table 2. The oral status of Cognitive study subjects was determined based on caries examinations (bordered by dashed lines in Table 2): low disease defined as >60% sound/filled teeth present; high disease defined as >40% teeth missing or decayed.

**Table 2:** Cognitive study clinical evaluations

Patient code	DT	DQ	DV	DAA	DZ
Age	74	93	77	91 y	77 y
Sex	F	F	M	F	F
Race	Afr/Am	White	White	White	White
Smoker	No	No	No	Yes	No
					
**Periodontal**					
(Number of teeth probed)	22	26	22	nd^1^	10
Probing depth (mean					
mm/site)	3.01 ± 0.53	1.98 ± 0.71	2.45 ± 0.55	nd	1.66 ± 0.41
Gingivitis (% positive sites)	68.2	4.5	9.1	nd	80
Calculus (% positive sites)	0	0	4.5	nd	30
					
**Caries**					
Dentures (lower)	0	Partial	0	0	0
Dentures (upper)	0	0	Partial	Partial	Full
Tooth index (% of 32 teeth)					
Sound	59.4	31.3	40.6	37.2	21.9
Missing	31.3	15.6	31.3	40.6	65.6
Filled	6.25	40.6	18.8	15.6	9.4
Decayed	3.1	0	0	6.3	3.1
Crown	0	12.5	9.4	0	0
					

^1^No periodontal examination data was acquired for DAA.

^2^Dashed lines border clinical parameters used to determine the oral status of Cognitive study participants.

### DNA extraction and 16S rRNA gene sequence analysis

DNA was extracted and purified from subgingival plaque using the UltraClean Soil DNA Isolation Kit (MO Bio Labs, Carlsbad, CA). The 16S rRNA gene was PCR amplified using the universal bacterial 16S rRNA primers (forward, 5'-GAGTTTGATYMTGGCTCAG, reverse, 5'-GAAGGAGGTGWTCCADCC [14]). Each PCR reaction contained 1 μl purified DNA, 0.4 μM universal forward and reverse primers, 5 μl 10X platinum PCR buffer, 1.5 mM MgSO_4_, 0.2 mM dNTPs and 0.5 μl Platinum TAQ DNA Polymerase High Fidelity (Invitrogen, Life Technologies Corp, Carlsbad, CA). PCR conditions were: 94°C for 4 minutes, followed by 30 cycles of 94°C for 45 seconds, 60°C for 45 seconds and 72°C for 90 seconds, with a final extension at 72°C for 15 minutes. PCR products were analyzed by 0.8% agarose gel electrophoresis, and reactions yielding products of ~1500 bp were cloned using the TOPO TA Cloning Kit for Sequencing (Invitrogen). Ligation products were electroporated into *Escherichia coli *(TOP10 Chemically Competent cells, Invitrogen), and transformants were incubated in 250 μl SOC medium (2% tryptone, 0.5% yeast extract, 10 mM NaCl, 2.5 mM KCl, 10 mM MgCl_2_, 10 mM MgSO_4_, 20 mM glucose) for 1 hour at 37°C, prior to plating on LB agar containing 50 μg/ml kanamycin and a 25 μl overlay of 2% X-gal. Following incubation overnight, ~100 colonies containing inserts (white colonies) were isolated from each sample and cultured at 37°C overnight in 96 well plates containing LB broth with 50 μg/ml kanamycin. A 2 μl volume of bacterial culture from each well was PCR amplified using M13 primers (forward, 5'-TGTAAAACGACGGCCAGT, reverse 5'-CAGGAAACAGCTATGAC). Reactions contained 0.2 μM of each primer, 5 μl 10X Qiagen PCR buffer (Qiagen, Valencia, CA), 0.2 mM dNTPs and 0.2 μl Taq DNA polymerase (Qiagen). PCR conditions were: 94°C for 4 minutes, followed by 30 cycles of 94°C for 45 seconds, 52°C for 45 seconds and 72°C for 90 seconds, with a final extension at 72°C for 15 minutes. PCR products from each well were examined by electrophoresis, and products of the appropriate size were sequenced by a commercial facility (SeqWright, Houston, TX).

### 16S rRNA gene sequence analysis

Each DNA sequence was scanned for a single segment of the original primer sequence (ATCAAACT) between bp 440 and 520 to identify the 16S rRNA gene and to help exclude chimeras. The initial part of each sequence containing uncalled bases (N) was removed, the distal part (vector sequence) was trimmed at the primer, and the sequence was reversed to standard orientation. Nucleotide sequences generated in this study have been deposited in GenBank, accession numbers HQ894465 - HQ895588.

Sequences were classified by BLASTN analysis against both a local database and the GenBank non-redundant (nr) database. The local database was assembled in an interative process: if a new experimental sequence did not match with >98% identity, a new database was assembled adding matching type sequences obtained from GenBank and the Ribosomal Database Project (RDP) [15]. The current local database contains 375 sequences organized into 122 'groups'. Each group contains closely related sequences and has been validated to be non-overlapping by BLAST analysis of individual groups against the entire database. This approach provided a level of detail that was informative for classifying bacterial sequences. Software used in sequence analyses included: 1) BLAST, using the NCBI server [16] with results formatted as XML; 2) UniFrac, using its server [17,18]; 3) phylogenetic tree construction, using the RDP server; 4) local BLAST, using the 'legacy' executable blastall from NCBI [19]. Other software run locally included: R [20] and the Analysis of Phylogenetics and Evolution (APE) package for constructing and plotting phylogenetic trees and plotting the Principal Coordinate Analysis (PCoA); ClustalW2 [21] and Muscle [22] for multiple sequence alignment; and Python [23] to automate steps in analyses and produce the heatmaps (generated using matplotlib).

### Human Oral Microbe Identification Microarray (HOMIM) analysis

For HOMIM 16S rRNA gene microarray analysis, purified DNA from subgingival plaque samples was sent to the HOMIM Core Facility at the Forsyth Institute (Boston, MA). This facility offers high throughput analysis of ~300 of the most prevalent oral bacterial species and provides a comprehensive report of bacterial profiles within DNA samples [24].

## Results and Discussion

### Identification of bacterial populations in subgingival plaque that differentiate individuals with high or low oral disease in West Virginia

Previous studies found bacterial species in the 'red complex' to be strongly linked to periodontal disease [25]. To examine if these same bacteria were associated with oral disease in West Virginia, we used 16S rRNA gene sequencing to characterize bacteria in subgingival plaque of COHRA participants diagnosed as having low or high oral disease based on periodontal examinations (Table 1, bordered by dashed lines). Analyses were performed in 96-well plate format, and due to the relatively high cost of DNA sequencing, our practical goal was to sequence 96 clones per sample, realizing that we would not be able to detect bacterial types present at low frequency. In actuality the number of clones sequenced per sample ranged from 55 to 133, with low numbers relating to difficulties obtaining high quality sequences for some samples. Sequences obtained were analyzed by BLAST against the GenBank database that includes all known bacterial 16S rRNA gene sequences, and against a local database that facilitated classification of bacterial 'types' based on ≥95% sequence identity. Subsequent evaluation of sequence data using the Human Oral Microbiome Database (HOMD) BLAST server [26] yielded nearly identical results, except for a few changes caused by reclassification of Clostridiales type strains, which affected some assignments to *Eubacterium *and Lachnospiraceae.

**Figure 1:**
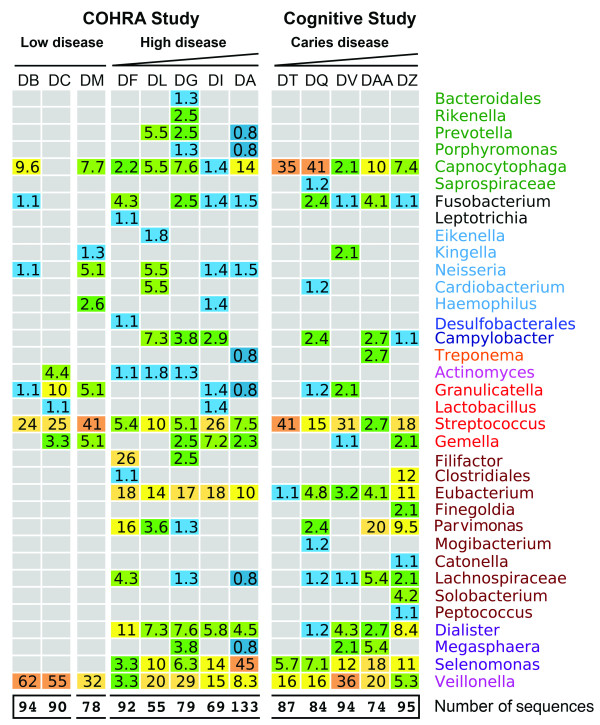
**Identification of bacterial populations in subgingival plaque of West Virginians**. Bacterial composition in plaque samples was determined using 16S rRNA gene sequencing in 2 low and 5 high oral disease COHRA participants, ranked based on periodontal exams, and 5 Cognitive study participants, ranked based on caries index. DM is a low periodontal disease control sample obtained through the West Virginia University Dental Clinic. Each numbered box indicates the percentage of clones of the type-specific bacterial 16S rRNA gene relative to the total number of clones sequenced, which is indicated at the bottom of each column. The color of the box reflects observed counts.

The initial phase of sequencing examined 2 low (DB and DC) and 5 high (DF, DL, DG, DI, DA) disease COHRA participants. An additional low disease sample (DM), evaluated based on periodontal criteria, was obtained through the West Virginia University Dental Clinic and served as a non-COHRA low disease control. The percentage of a bacterial type in each sample relative to the total number of clones sequenced (indicated at the bottom of each column) is shown in the heatmap in Figure 1. As previously reported [6], increased bacterial diversity was evident in plaque from individuals diagnosed with high oral disease. However, unexpectedly a large number of high disease bacterial types were classified in the Clostridiales cluster (*Selenomonas*, *Eubacterium*, *Dialister*), which were completely absent from low disease plaque. Also notable in high disease was the low frequency of bacterial types traditionally linked to periodontitis (*Porphyromonas*, *Tannerella*, *Treponema *and *Aggregatibacter*). Plaque from low disease exhibited a distinctly different bacterial population, including mainly *Streptococcus *and *Veillonella *with a moderate number of *Capnocytophaga*. The bacterial diversity in low disease COHRA samples was highly consistent with a recent study, which identified *Streptococcus*, *Veillonella *and *Capnocytophaga *in 100% of the plaque samples obtained from individuals with healthy oral cavities [27]. Thus, while bacteria associated with oral health in West Virginia was highly reflective of other regions in the United States, an atypical pathogenic phylogenetic signature that includes *Selenomonas *and other Clostridiales cluster bacteria appeared to be associated with the decline in oral health in West Virginia.

COHRA study participants ranged in age from 23 to 48, and Cognitive study participants (ages 73 to 93) provided a means of assessing how bacterial types related to tooth status later in life. The periodontal health of the 5 Cognitive study participants included in this study was exceptionally good, as evident in probing depths of 3 mm or less, shown in Table 2. However, as expected for this age, considerable variation was observed in caries health (Table 2, bordered by dashed lines), with DT having the highest percentage of sound teeth, and DAA and DZ having the highest percentage of missing/decayed teeth. Examination of bacterial types in subgingival plaque of DT revealed mainly *Veillonella*, *Streptococcus *and *Capnocytophaga *(Figure 1), as observed for low disease COHRA participants. In comparison, DAA and DZ had bacterial patterns that included increased diversity and species richness of *Selenomonas*, *Eubacterium *and *Dialister*, in addition to many of the other bacterial species observed in high oral disease COHRA participants. These data are consistent with the bacterial signature of aged West Virginians with tooth loss being similar to that of younger West Virginians with periodontal disease.

### Statistical analyses of bacterial types identified in subgingival plaque

**Figure 2:**
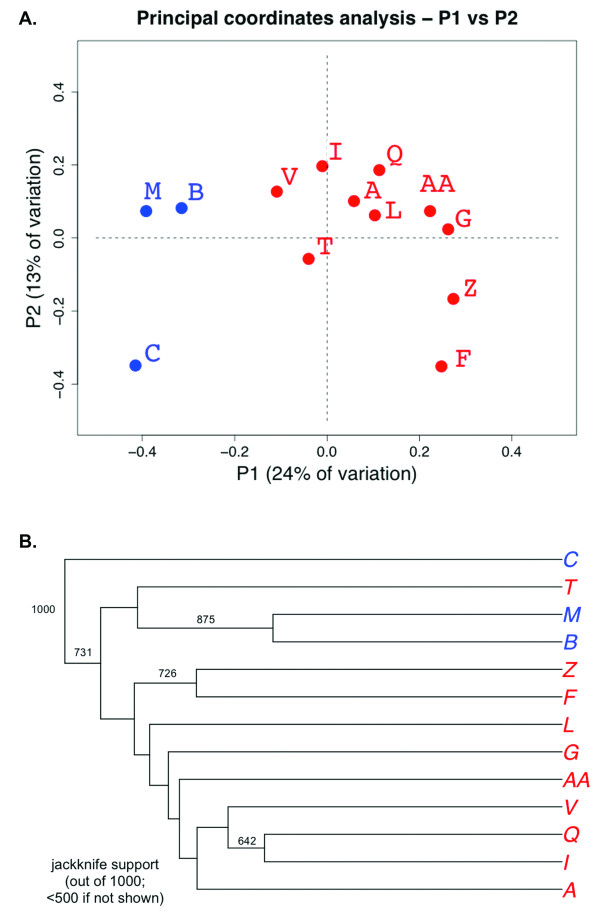
**Statistical analyses of bacterial populations in low and high disease plaque**. A) Principal coordinate analysis of bacterial communities from subgingival plaque of West Virginians with low oral disease (blue) as compared to plaque of West Virginians with varying degrees of oral disease (red). B) The Unifrac algorithm was used to compute the unique branch length for a given sub-sample. Cluster analysis split low (blue) and diseased samples (red) into different clades. Support for the clusters was evaluated by jackknife tests (1000 replicates). T was the least diseased sample in the Cognitive study, as ranked in Table 2. The 'D' has been removed from sample notations in these figures for clarity.

Unifrac analysis [17], which takes into account phylogenetic distances, has become the method of choice for analyzing differences between microbial communities and was used in our studies to provide statistical validation of differences in low and high disease plaque environments. The method requires a rooted phylogenetic tree as input, and we used the RDP server to generate the tree, which is especially suited for rRNA alignments as it employs Infernal [28] that is guided by predicted secondary structure. The tree was constructed by the neighbor-joining method [29], using APE in R. It was rooted by including *Thermotoga maritima *SL7 as an outgroup. The Unifrac algorithm was used to compute the unique fraction of branch length for each sample. These results (which are phylogenetic distances separating the individual samples) were analyzed using cluster analysis (UPGMA) and PCoA and ignored duplicate sequences. As shown in Figure 2A, PCoA separated low disease from diseased samples into two clusters along the first eigenvector. Cluster analysis also split low disease and diseased samples into different clades, with sample DT (labeled T in Figure 2), the least diseased sample based on caries outcome in the Cognitive study, splitting to the low disease clade. Support for the clusters was evaluated by jackknife tests (1000 replicates; Figure 2B). When analyses were again performed with all the sequences relabeled as diseased or healthy, Unifrac analysis found the two environments to be statistically different (*P *< 0.001), whether or not duplicate sequences were considered.

### Analysis of the bacterial populations in plaque using HOMIM analyses

**Figure 3:**
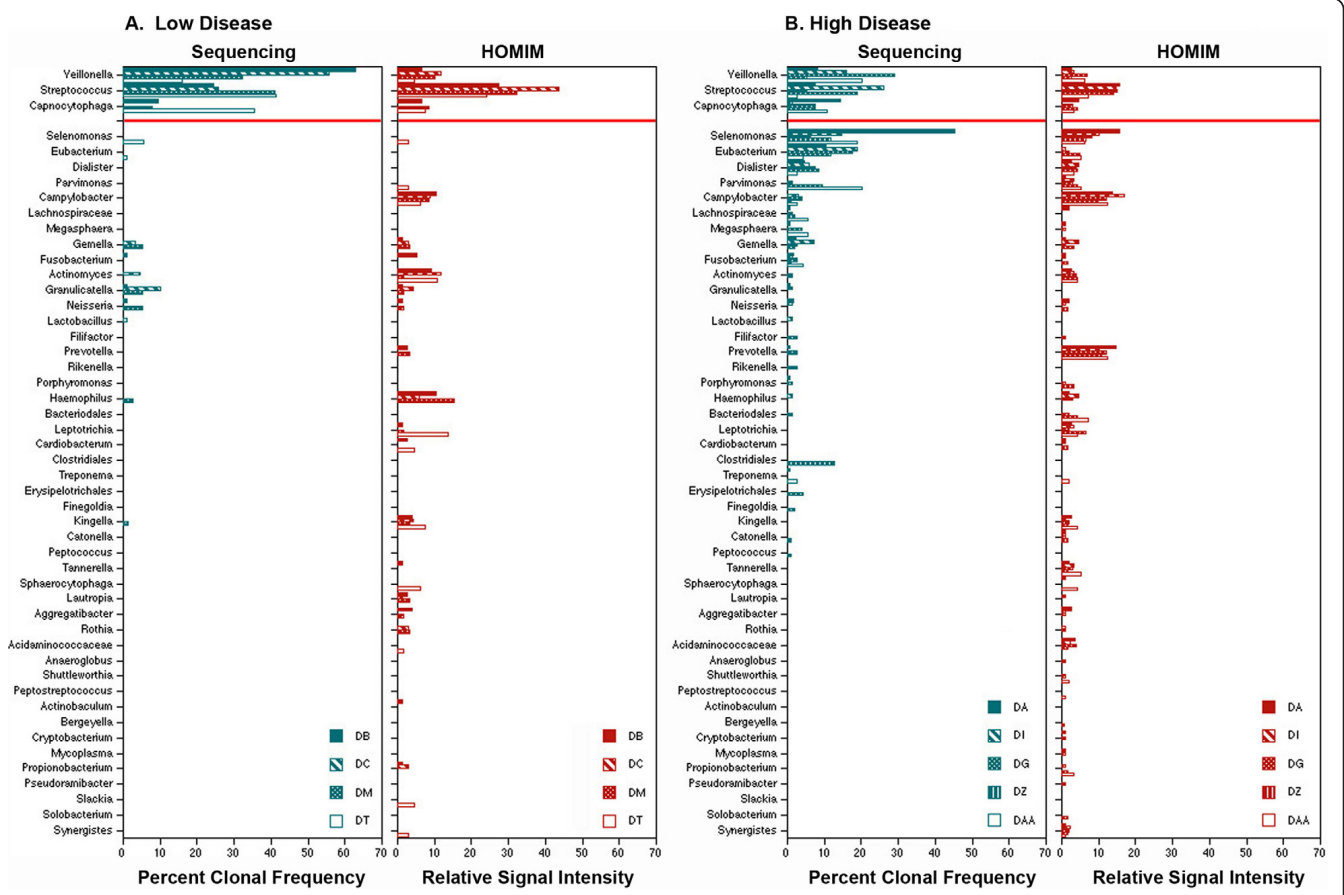
**Comparison of 16S rRNA gene analyses using sequencing and HOMIM**. The frequency of bacterial types (by percentage), determined by 16S rRNA sequencing, of 4 plaque samples from low disease and 5 plaque samples from high disease West Virginians, ranked based on criteria defined in Materials and Methods, was compared with the microarray signal intensity obtained from the same samples in HOMIM analyses. Bacterial types above the red line were more frequent in low disease.

Since rRNA gene sequencing revealed an unexpected bacterial signature in plaque of West Virginians with high oral disease, we asked whether this pattern could be detected using an alternative method of 16S rRNA gene analysis, HOMIM. In HOMIM, multiple primers are initially used to amplify 16S rRNA genes within a DNA sample, which are then assayed for the presence of specific 16S rRNA genes using probes designed to optimize detection of 272 different bacterial species [30]. Unlike 16S rRNA sequencing, which allows quantification of the frequency of bacterial clones, the read-out for HOMIM is a relative signal intensity for each detected 16S rRNA sequence on a scale from 0 to 5. In our studies HOMIM results are presented as the sum of the signals for all oral taxa within each genus. Comparisons of HOMIM and DNA sequence analyses of plaque samples from 4 low disease (DB, DC, DM, DT) and the 5 highest disease (DA, DI, DG, DZ, DAA) participants in our study are shown in Figure 3. HOMIM analyses of plaque from low disease detected a high frequency of *Veillonella*, *Streptococcus *and *Capnocytophaga*, closely paralleling results of 16S rRNA sequencing (Figure 3A). Less prevalent bacterial types detected in 16S rRNA sequencing, such as *Gemella*, *Fusobacterium*, *Actinomyces*, *Granulicatella*, *Neisseria *and *Haemophilus*, were confirmed by HOMIM. Additional bacterial types, presumably at too low a frequency to detect by sequencing, were also detected in low disease by HOMIM, the most evident of which were *Campylobacter*, *Prevotella*, *Leptotrichia*, *Lautropia *and *Aggregatibacter*. These results highlight differences that can be observed between DNA sequencing and HOMIM due to methodologies employed in HOMIM that can increase the sensitivity of detection of specific bacterial species by 10 fold.

In plaque from individuals with high disease, HOMIM analyses confirmed the presence of genera from the Clostridiales order, including *Selenomonas*, *Eubacterium *and *Dialister *(Figure 3B). Notably, as with sequencing data, bacteria in the 'red complex' were either absent or present at low levels in HOMIM analyses. As with low disease plaque, disproportionately higher signals for certain bacteria, specifically *Campylobacter*, *Prevotella *and *Leptotrichia*, were observed in HOMIM analysis as compared with sequencing, again explained by the increased sensitivity of microarray detection methodologies. Importantly, conclusions from HOMIM analyses confirmed those from 16S rRNA sequencing, and identified increased genus richness and high intensity signals for *Selenomonas*, *Eubacterium *and *Dialister *in association with a decline in oral health.

### Origin of bacterial types in high disease plaque

**Figure 4:**
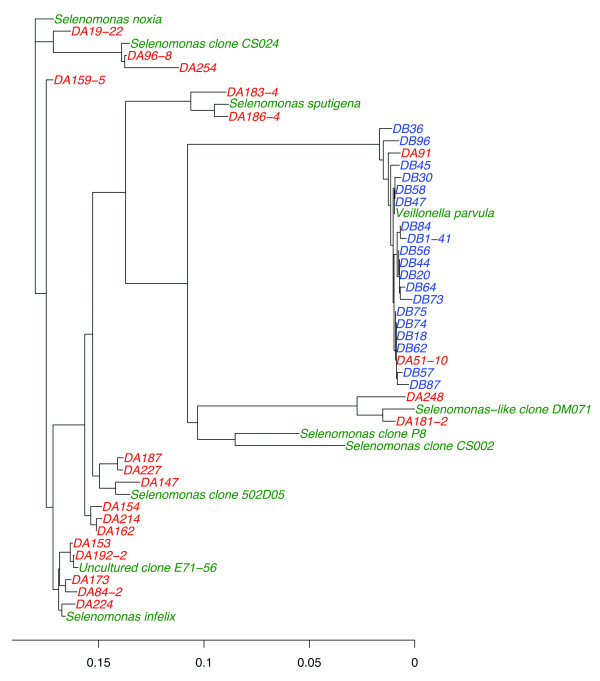
**Phylogenetic trees of *Selenomonas *and *Veillonella *isolated from individual plaque samples**. 16S rRNA gene sequences from a single high disease plaque sample (DA, red text) and a single low disease plaque sample (DB, blue text) were used to generate phylogenetic trees of *Selenomonas *and *Veillonella*, respectively. The x-axis indicates percent difference in 16S rRNA gene sequence. The separated clusters of *Selenomonas *show that this population descended from independently colonizing but phylogenetically related bacteria. *Veillonella *from DB exhibited limited diversity.

Phylogenetic trees of bacterial types developed using 16S rRNA sequencing also provided insight into the origin of bacterial signatures associated with high oral disease. For example, comparisons of 16S rRNA gene sequences revealed that diverse *Selenomonas *spp. were actually repeated colonizers of the high disease oral environment. The 16S rRNA tree represented in Figure 4 shows the broad diversity of *Selenomonas *phylotypes recovered in a single plaque sample from a high disease subject (DA). In contrast, the same clade in a sample from a low disease subject (DB) contained only low diversity *Veillonella *phylotypes. The order Clostridiales has its phylogenetic assignment in the Firmicutes (typically Gram-positive), but it includes a large family, the Veillonellaceae, which are obligate anaerobes that stain Gram-negative due to a porous pseudo-outer membrane. Low disease *Veillonella *have nearly identical sequences typical of a single species (> 97% 16S rRNA gene sequence identity), as represented in Figure 4. In our studies, we found the tight cluster of *Veillonella *observed in low disease was replaced in plaque from high disease by a broad expansion of other members of the family Veillonellaceae, such as *Selenomonas *and *Dialister*. At the same time in high disease there was an expansion of Gram-positives from the order Clostridiales, primarily in the families Eubacteriaceae and Lachnospiraceae. The breadth of this bacterial group is large (minimum ~85% identity), and this allowed us to recognize that Clostridiales were repeated colonizers in plaque of individuals having high oral disease, as represented by the phylogenic diversity of *Selenomonas *in Figure 4. This finding is significant because it highlights the role of the oral environment (as opposed to in situ evolution due to horizontal gene transfer) in the generation of bacterial signatures associated with high oral disease in West Virginia.

## Conclusions

The etiology of the poor oral health leading to the high tooth loss statistics in West Virginia is unknown. The goal of this study was to gain insight into this problem using 16S rRNA gene analyses to characterize the bacterial species in subgingival plaque of West Virginians having high oral disease. The most striking finding of our analyses was the predominance of Clostridiales cluster bacteria, including genera such as *Selenomonas*, *Eubacterium *and *Dialister*, in plaque of West Virginians having high oral disease. The oral microbiota is complex and dynamic, which compromises conclusions derived from this preliminary study, but factors that strengthen our interpretation that an atypical bacterial signature contributes to the oral health disparity in West Virginia include: 1) characterization of similar microbiological patterns in high and low disease plaque using of two methods of 16S rRNA gene analysis, and 2) detection of a similar relationship between bacterial types and oral disease in two separate West Virginia populations. The breadth of the Clostridiales also allowed us to recognize that bacteria linked to oral disease were repeated colonizers of individuals with high disease, suggesting a functional relationship between the disease environment and these disease-associated bacteria. The possibility that an atypical bacterial phylogenetic signature might be linked to the decline in oral health in West Virginia is a novel idea and may prove integral to understanding the etiology of the dental problems in this state. The results from this pilot project highlight the need for an expanded study that uses culture independent approaches to analyze bacterial populations in a larger number of West Virginians having low or high oral disease to confirm the relationship between bacterial profiles and disease. The knowledge derived from such a study has the potential of being applied to the development of targeted intervention strategies that modify environmental factors to preclude colonization by disease-associated bacteria.

## List of Abbreviations

APE: Analysis of Phylogenetics and Evolution; BOP: bleeding on probing; COHRA: Center for Oral Health Research in Appalachia; HOMIM: Human Oral Microbe Identification Microarray; HOMD: Human Oral Microbiome Database; NHANES: National Health and Nutrition Examination Survey; nr: non-redundant; PCoA: Principal Coordinate Analysis; PD: probing depth; RDP: Ribosomal Database Project; rRNA: ribosomal RNA; UPGMA: Unweighted Pair Group Method with Arithmetic Mean; WVU: West Virginia University.

## Competing interests

BJP is the director of the HOMIM Core Facility at The Forsyth Institute, which performed the bacterial microarray analyses.

## Authors' contributions

JCO organized the project and drafted the manuscript. CFC assisted with project organization and drafting of the manuscript. SL developed 16S rRNA gene amplification and cloning methods. EL performed 16S rRNA gene amplification and cloning. YC organized Cognitive study clinical data. BW organized acquisition of specimens and clinical data for the Cognitive study. RJC organized the alignment of our project with the COHRA and Cognitive studies and assisted with clinical analyses and project development. JGT organized and facilitated acquisition of COHRA subgingival plaque samples. DWM assisted with the design of the COHRA and Cognitive studies and with the acquisition of clinical samples. RJW and MLM assisted with the design of the COHRA project and organization of clinical data. BJP performed HOMIM analyses and helped with developing methods and analyses of 16S rRNA gene sequences. TE performed all 16S rRNA sequence data analyses. All authors read and approved the final manuscript.

## Pre-publication history

The pre-publication history for this paper can be accessed here:

http://www.biomedcentral.com/1472-6831/11/7/prepub
